# The underlying processes of a soil mite metacommunity on a small scale

**DOI:** 10.1371/journal.pone.0176828

**Published:** 2017-05-08

**Authors:** Chengxu Dong, Meixiang Gao, Chuanwei Guo, Lin Lin, Donghui Wu, Limin Zhang

**Affiliations:** 1 College of Geographical Sciences, Harbin Normal University, Harbin,China; 2 Key Laboratory of Remote Sensing Monitoring of Geographic Environment, College of Heilongjiang Province, Harbin Normal University, Harbin, China; 3 Key laboratory of Wetland Ecology and Environment, Northeast Institute of Geography and Agroecology, Chinese Academy of Sciences, Changchun, China; Nanjing Agricultural University, CHINA

## Abstract

Metacommunity theory provides an understanding of how ecological processes regulate local community assemblies. However, few field studies have evaluated the underlying mechanisms of a metacommunity on a small scale through revealing the relative roles of spatial and environmental filtering in structuring local community composition. Based on a spatially explicit sampling design in 2012 and 2013, this study aims to evaluate the underlying processes of a soil mite metacommunity on a small spatial scale (50 m) in a temperate deciduous forest located at the Maoershan Ecosystem Research Station, Northeast China. Moran’s eigenvector maps (MEMs) were used to model independent spatial variables. The relative importance of spatial (including trend variables, i.e., geographical coordinates, and broad- and fine-scale spatial variables) and environmental factors in driving the soil mite metacommunity was determined by variation partitioning. Mantel and partial Mantel tests and a redundancy analysis (RDA) were also used to identify the relative contributions of spatial and environmental variables. The results of variation partitioning suggested that the relatively large and significant variance was a result of spatial variables (including broad- and fine-scale spatial variables and trend), indicating the importance of dispersal limitation and autocorrelation processes. The significant contribution of environmental variables was detected in 2012 based on a partial Mantel test, and soil moisture and soil organic matter were especially important for the soil mite metacommunity composition in both years. The study suggested that the soil mite metacommunity was primarily regulated by dispersal limitation due to broad-scale and neutral biotic processes at a fine-scale and that environmental filtering might be of subordinate importance. In conclusion, a combination of metacommunity perspectives between neutral and species sorting theories was suggested to be important in the observed structure of the soil mite metacommunity at the studied small scale.

## Introduction

An important goal of community ecology is identifying what drives community assembly and biodiversity maintenance across different scales [1,2]. However, it is difficult to answer the question clearly, as the structures and patterns of communities are complicated and have significant spatial and temporal variance [3]. Recently, metacommunity theories have been increasingly used to identify the underlying processes that construct community assemblies for different organisms.

The metacommunity perspectives recognize the mechanisms that affect distribution, as they correlate to the local community structure [4]. Four metacommunity perspectives have been used to explain variations in a local community structure, including the neutral model, species sorting, mass effects and patch dynamics. From a neutral perspective, species do not differ in their niche and fitness [5]. Dispersal limitation, random speciation, ecological drift and migration determine the local community composition [5]. From a species-sorting perspective, both biotic interactions and environmental variation filter species co-existence at each locality [6]. Sufficient dispersal allows for the environmental variability of various species among different sites. From the mass-effects perspective, high dispersal rates allow species to exist at sites with unsuitable environmental conditions. Thus, both dispersal and environmental variables are suggested to be important. From a patch-dynamics perspective, co-occurring species differ through being either good competitors or good colonizers within a homogeneous habitat [6–8]. Although the species-sorting and mass-effects perspectives are the most commonly evaluated and supported perspectives [9], the view that the four perspectives may function simultaneously in a metacommunity has been suggested [10,11]. Moreover, Winegardner et al. [10] suggested that ecologists should break down these perspectives and pay attention to the environmental and dispersal effects on metacommunity structuring, which are fundamental organizing processes in all metacommunities [3,12]. Therefore, disentangling the relative contributions of environmental and spatial processes in a metacommunity has been suggested to evaluate the four perspectives [9,11]. Moreover, spatial patterns of organisms are scale-dependent, and different perspectives may regulate biodiversity at different spatial scales [13,14]. However, in contrast to regional or local scale field experiments [15,16], metacommunity theories at small scales (10^1^–10^3^ m) [17] remain unclear, especially for small animals inhabiting belowground ecosystems.

Soil mites are a major group of wingless microarthropods living in temperate forests [18], but little is known about the relative roles of spatial and environmental processes on soil mite metacommunity structure on a small scale. Both species sorting and mass effects have been suggested to apply to the soil mite metacommunity structure at the regional scale [19], while a species-sorting process was possibly detected for a soil mite metacommunity in a “mainland-island” manipulated experiment at a small scale [20]. Although spatial accessibility is usually easier to obtain at a small scale, we assumed that dispersal limitation should be an important driver for shaping a soil mite metacommunity because these animals are not highly mobile within the soil. It has been suggested that soil mites might be sensitive to environmental change due to their low rate of reproduction and long life cycle [21]. Soil environmental conditions are usually variable and influence earthworm community composition on a small scale [22]. Therefore, we hypothesized that environmental filtering is also an important driver of soil mite metacommunity structure. Thus, we hypothesized that the neutral and species-sorting perspectives should be controlling the soil mite metacommunity simultaneously on a small scale.

In this study, a soil mite metacommunity was selected on a small scale (50 m) in a temperate forest of Northeast China. In this experiment, the totality of the samples within the plot represent a metacommunity, which is defined as “a set of ecological communities at different sites that are linked by the dispersal of multiple, potentially interacting, species” [23–25]. A local community is represented by, “a group of species at a given site (i.e., the sample within the plot)” [25]. This study aims to quantify the relative contributions of spatial and environmental variables as determinants of a soil mite metacommunity structure and to recognize the relative importance of metacommunity theories at a small scale (50 m).

## Materials and methods

### Description of study site

The study was performed at the Maoershan Ecosystem Research Station (127°30′–34′E, 45°20′–25′N) of the Northeast Forestry University in Heilongjiang Province, China. The forest in this region is considered to be a typical forest of northeastern China. The average altitude is approximately 300 m. The parent material is granite bedrock. The soil is Hap-Boric Luvisol [26]. The average slope is approximately 10–15°. The research area is characterized by a continental temperate monsoon climate, which is dry and cold in the winter and warm and humid in the summer. The annual precipitation is approximately 600–800 mm, of which 80% occurs in July and August. The annual evaporation is approximately 884 mm. The mean annual, January and July air temperatures are 2.8°C, -31°C and 32°C, respectively. There are approximately 120–140 frost-free days, with an early and late frost in September and May, respectively.

The soil mite metacommunity was sampled in a temperate deciduous forest at the Maoershan Ecosystem Research Station. The field experiments were permited by the office of Maoershan Ecosystem Research Station. The field studies did not involve endangered or protected species. The sampling site was located within a secondary forest that was greater than 60 yr old. The dominant tree species were *Ulmus davidiana* var. *japonica*, *Fraxinus mandshurica*, *Betula platyphylla*, *Populus davidiana*, *Juglans mandshurica*, *Acer mono*, *Tilia amurensis* and *Populus ussuriensis*. The dominant shrub species were *Syringa reticulata* var. *amurensis*, *Padus racemosa*, *Acer ginnala* and *Corylus mandshurica*.

### Collecting the soil mite metacommunity and soil samples

One experimental plot (50× 50 m^2^) was established at the study site in August 2012. The plot was equally divided into 100 squares of 5 × 5 m^2^, with 121 nodes in the plot. Soil samples without a litter layer were collected near each node. A node was located near the bottom left corner of each square. Squares (15 × 15 cm^2^ and 10 cm depth) and cylindrical soil cores were sampled using a soil auger (7-cm diameter and 10 cm depth). These samples were collected to extract the soil mite metacommunity in August in 2012 and 2013, respectively. Soil mite communities were removed from the collected soil samples using the Berlese-Tullgren method. The soil mite communities were then preserved in a 95% alcohol solution. Only the adult soil mites were identified and counted at the species level [27–30]. Juvenile soil mites were excluded from all analyses.

Litter-free soil samples (squares with 5 × 5 cm^2^ and 10 cm depth in 2012, and cylinders with a 7-cm diameter and 10-cm depth in 2013) were extracted directly to the right of the location where the soil mite communities were collected. Soil samples were air-dried and sieved to 1 mm. After digestion of the samples in H_2_SO_4_, the colorimetric method was used to obtain the soil organic matter content (SOM, gkg^-1^). The soil pH was measured indeionized water with a soil/solution ratio of 1:5. The soil water content (SWC, %) was determined gravimetrically [31]. The litter dry weight (LDW, g) and litter water content (LWC, %) were also investigated.

### Statistical analysis

The species abundance matrix was standardized using Hellinger distances before analysis. A square root transformation of the resulting values was used to control the influence of large abundance values [32]. A significant linear trend in the soil mite metacommunity was detected in 2012 and in 2013.The linear trends were surrogates of spatial patterns acting at a broader scale than the sampling extent [33], which was considered as a source of variation along with the other explanatory variables [32]. Thus, the species abundance matrix was not detrended before performing variation partitioning. The X- and Y-coordinates were independently forward selected prior to variation partitioning. This method used both a significance level of 0.05 and (adjusted-*R*^2^) as double stopping criteria [34]. The selected significant coordinates were saved as a new object and then incorporated into the partitioning procedure.

Spatial structure can be modeled according to a set of independent component patterns (Moran’s eigenvector maps, MEMs) [35], formerly called PCNM (Principal Coordinates of Neighbor Matrices). Those spatial variables (MEMs) were calculated from a spectral decomposition of a truncated distance matrix of the spatial relationships among the sampling sites in this study, which correspond to a general sequence of broad- to fine-scale variations within a given spatial extent. The MEMs approach models *n*-1 spatial variables with positive and negative eigenvalues [32]. Only those MEMs with positive eigenvalues were selected as spatial explanatory variables, as those valuables are agents of contagious processes that are frequently observed in natural ecosystems [32]. Then, a forward selection procedure was conducted to select MEMs that had obvious effects on the species matrix [34]. Among the selected MEMs, eigenvectors related to high eigenvalues express broad-scale patterns of relationships among sampling sites, whereas those related to low eigenvalues express fine-scale patterns [32,36]. According to the scales of the patterns that the significant MEMs represent (as collected from the maps of the significant MEMs, S2 and S3 Figs), the final selected MEMs were ranked in descending order based on their eigenvalues. MEMs with large eigenvalues were assigned to the broad-scale fraction, while MEMs with smaller eigenvalues were assigned to the fine-scale fraction [32].

The environmental contributions were identified based on the following five variables: pH, SWC, LWC, LDW and SOM. Those environmental variables were considered to be important factors shaping and influencing community structure [22,37,38]. To adjust for collinearity, the five environmental factors were subjected to a principal component analysis (PCA). Then, both a redundancy analysis (RDA) and linear regression were used to discriminate whether the spatially structured variations of the soil mite metacommunity were significantly related to the specific environmental principal components (PCs) on broad and fine scales [32].

Finally, the subset of coordinates (trend variables), significant MEMs (including broad- and fine-scale fractions) and environmental variables (PCs) were used as explanatory variables in variation partitioning. This approach was applied to recognize the relative importance of environmental and spatial filtering to the soil mite metacommunity structure [38]. The amount of variation in community composition due to these processes was partitioned by performing a partial redundancy analysis (pRDA). The significance of each source of variation was evaluated with a Monte Carlo permutation test (999 permutations).

To further explain the relative roles of environmental and spatial variables in the soil mite metacommunity composition, a Mantel test, partial Mantel test and a redundancy analysis (RDA) were used. A Mantel test was applied to examine the possible association between the soil mite metacommunity and spatial, environmental factors. Before the Mantel test was performed, the abundance data of the soil mite metacommunity were transformed with a Hellinger transformation. A Bray-Curtis dissimilarity index was calculated to examine the dissimilarity of the soil mite communities between sites. Dissimilarity matrices of spatial and environmental data sets were calculated based on Euclidean distance after *z*-transformation. A Spearman correlation coefficient was applied, and the significance level was examined using a permutation method with 999 repetitions. Next, a partial Mantel test was also performed to examine correlations among the soil mite metacommunity dissimilarity, ecological and geographical distances. Then, a redundancy analysis (RDA) was used to further assess which environmental variables (PCs) contributed most of the variation in the soil mite metacommunity composition.

The multivariate analyses were implemented using the “vegan” [39],“PCNM” [40]and “packfor” packages [41] in R software, version 3.1.2.

## Results

### Identification of a soil mite metacommunity and descriptions of the environmental, trend and spatial variables

In August 2012 and 2013, 19 and 18 species were detected and 16240 and 8331 individuals were caught, respectively. *Scheloribates* sp., *Pachyseius* sp., *Gamasolaelaps* sp., *Eulohmannia* sp. and *Nanhermannia* sp. were relatively abundant and widely distributed in both 2012 and 2013 (Table 1). The sampling effort was enough to reveal the overall richness of the soil mite metacommunity for each year according to the sample-based rarefaction curves [42] (S1 Fig).

**Table 1:** Species and individuals of soil mite metacommunities in 2012 and 2013.

		2012				2013		
Species	Individuals ^a^	Percentage (%)	Coefficient of variation (%)	Frequency ^b^	Individuals ^a^	Percentage (%)	Coefficient of variation (%)	Frequency ^b^
*Macrocheles* sp.	809	4.98	109.67	E	263	3.16	171.08	C
*Pachyseius* sp.	1 568	9.66	83.82	E	784	9.41	106.87	E
Epicriidae sp.	869	5.35	114.04	E	493	5.92	131.09	E
*Gamasolaelaps* sp.	1 487	9.16	124.33	E	675	8.10	133.04	D
*Nanhermannia* sp.	1 042	6.42	104.63	E	535	6.42	71.80	E
*Eulohmannia* sp.	1 428	8.79	103.20	E	812	9.75	114.45	D
*Belba* sp. 1	709	4.37	94.05	E	447	5.37	98.03	D
*Scheloribates* sp.	3 677	22.64	66.54	E	1 663	19.96	49.98	E
*Suctobelbella* sp.	785	4.83	140.84	E	447	5.37	98.03	D
*Geholaspis* sp.	403	2.48	103.89	D	197	2.36	302.27	C
*Protoribates* sp.	627	3.86	129.02	D	352	4.23	144.82	D
Oribatida sp.	548	3.37	123.67	E	294	3.53	130.45	D
*Acrotritia ardua*	660	4.06	90.37	E	310	3.72	128.46	C
Prostigmata sp.	319	1.96	209.37	C	152	1.82	266.12	B
*Ceratozetes* sp.	735	4.53	87.12	E	466	5.59	97.08	D
*Holaspulus* sp.	225	1.39	144.69	C	180	2.16	154.05	C
*Belba* sp.2	283	1.74	168.02	D	243	2.92	143.67	C
*Hypochthonius* sp.	63	0.39	319.56	A	18	0.22	320.76	A
Trombidiidae sp.	3	0.02	817.15	A	NF ^c^			NF

^a ^In 2012, a soil mite metacommunity was collected using square soil cores with 15 × 15 cm^2^ and 10 cm depth. In 2013, a soil mite metacommunity was collected using cylindrical soil cores and a soil auger with a 7-cm diameter and 10 cm depth.

^b ^Raunkiaer’s frequency class. A: 1–20%; B: 21–40%; C: 41–60%; D: 61–80%; E: 81–100%.

^c ^NF represents not found.

The environmental conditions are shown in S1 Table. The first four axes explained 87.19% of the total variation in 2012, in which the contributions of PC1, PC2, PC3 and PC4 were 30.23, 24.63, 18 and 14.33%, respectively. LWC, SWC, SOM and pH contributed the most to PC1, PC2, PC3 and PC4, respectively, in 2012. In 2013, the first four axes explained 86.53% of the total variation, there into the explanation of the PC1, PC2, PC3 and PC4 was 28.64, 22.43, 19.58 and 15.88%, respectively. LWC, LDW, SOM and SWC contributed the most to PC1, PC2, PC3 and PC4, respectively.

A significant linear trend in metacommunity composition was detected in 2012 and 2013. The X- and Y-coordinate significantly explained 5.03% and 6.22% of the variation, respectively.

A total of 15 MEMs (S2 Fig) were selected, and they explained 20.05% of the variation in the 2012 abundance data. Based on the scales of the spatial patterns relative to the significant MEMs, the MEM components # 1, 2, 3, 4, 5, 8, 9, 11, 12, 13, 14 and 19 were related to high eigenvalues and were defined as broad-scale. The MEM components # 25, 38 and 44 were related to low eigenvalues and were defined as fine-scale. In 2013, the 15 selected MEMs (S3 Fig) explained 19.12% of the variation. MEM components # 1, 2, 3, 4, 5, 8, 9, 11, 12, 13, 17 and 25 were classified as broad scale, and MEM components # 27, 28 and 43 were classified as fine scale.

### Relative roles of the spatial and environmental variables

**Fig 1:**
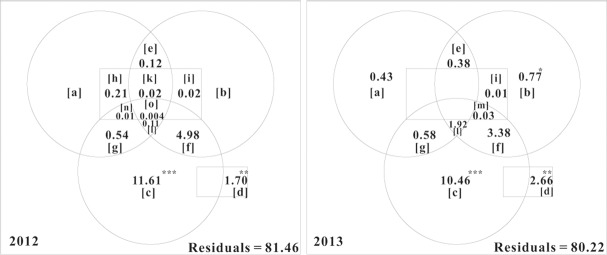
Variation partitioning (%) for the soil mite metacommunity estimated by partial redundancy analysis (pRDA). Fraction [a] means that variation explained by the purely environmental variables. Fraction [b] indicates that variation explained by the purely trend variables (geographical coordinates). Fraction [c] represents that variation explained by purely broad-scale MEMs. Fraction [d] means that variation explained by purely fine-scale MEMs. Fraction [g] is the common that was jointly explained by the environmental variables and broad-scale MEMs. [l] is the common fraction that was jointly explained by the environmental factors, trend variables and broad-scale MEMs. Fraction [h+k+n+o] means the common fraction that was jointly explained by the environmental factors and fine-scale MEMs. The small negative values were not labeled. * *P*<0.05. ** *P*<0.01. *** *P*<0.001.

In 2012, 18.54% of the variation in the soil mite metacommunity was explained, in which the significantly unique contributions of broad-scale [c] and fine-scale [d] variables were 11.61% and 1.70%, respectively. A very small negative *R*^2^_adj_ appeared for pure environmental [a] and trend [b] variables, implying that these explanatory variables explained less of the response variable’s variation than would be expected by chance. In 2013, 19.78% of the variation in the soil mite metacommunity was explained, in which the significantly unique contributions of trend [b], broad-scale [c] and fine-scale [d] variables were 0.77%, 10.46% and 2.66%, respectively. Pure environmental [a] variables uniquely contributed 0.43%. The common fractions corresponding to the environmental variables and broad-scale MEMs [g], the environmental variables, trend variables and broad-scale MEMs [l] and the environmental variables and fine-scale MEMs [h+k+n+o] were relatively low in both years (Fig 1).

The measured environmental variables in 2012 (PC1, PC2, PC3 and PC4) and in 2013 (PC1, PC2, PC3 and PC4) were significantly related to the broad-scale MEMs. No environmental factor was related to the fine-scale MEMs in both years (S2 Table).

Based on a Mantel test, there was a positive correlation between soil mite metacommunity composition and spatial factors in each year, while the correlation with environmental factors was not significant (Table 2). However, no significant association between the dissimilarity of the soil mite metacommunity and spatial distance was detected after controlling for covariance with environmental factors according to a partial Mantel test. A positive association between the dissimilarity of the soil mite metacommunity and environmental distance was found after controlling for the covariance with spatial factors (Table 2). Furthermore, the results of the RDA illustrated that the composition of the soil mite metacommunity in 2012 was influenced by PC1 and PC3 and in 2013 was influenced by PC1, PC3 and PC4 (Table 3).

**Table 2:** Simple and partial Mantel tests of soil mite metacommunity dissimilarity against spatial and environmental distances (999 permutations).

	2012		2013	
Mantel/Partial Mantel tests	*r*	*P*	*r*	*P*
Environment	-0.003	0.53	0.02	0.34
Space	0.11	<0.001***	0.15	<0.001***
Environment|Space ^a^	0.11	0.04*	0.04	0.25
Space|Environment ^b^	0.05	0.12	0.05	0.13

^a^ Soil mite community dissimilarity with environmental distance, controlling for spatial distance.

^b^ Soil mite community dissimilarity with spatial distance, controlling for environmental distance.

* *P*<0.05.

*** *P*<0.001.

**Table 3:** The effect of environmental factors on the soil mite metacommunity structures analyzed by redundancy analysis and Monte Carlo permutation test (999 permutations)

Factor	2012		2013	
	*R*^2^	*P*	*R*^2^	*P*
PC1 ^a^	0.14	<0.001***	0.11	<0.001***
PC2	0.01	0.61	0.01	0.43
PC3	0.06	0.03*	0.14	<0.001***
PC4	0.04	0.07	0.07	0.02*

^a^ PC indicates each of the factors that were obtained from the PCA for each of the data sets.

* *P*<0.05.

*** *P*<0.001.

## Discussion

The results of variation partitioning at a global spatial scale indicated that spatial variables, including both MEMs and trend (geographical coordinates), make significant and relatively large contributions to soil mite metacommunity composition. At the same time, according to the significant associations between spatial distances and soil mite metacommunities based on a Mantel test, the relative contribution of spatial processes to metacommunity structure was important. Geographical patterns (trend) in the soil mite metacommunity may arise from dispersal-related processes, such as regional historical events or climatic variables [43]. We found that the trend accounted for a significant fraction of variation in 2013, suggesting that the spatial structures resulting from a broader scale than the experimental extent (50 m) cannot be negligible [33]. Moreover, it is usually suggested that dispersal limitation is important at a larger scale, as significant geographic barriers could limit organisms’ dispersal and then isolate species into different metacommunities [44]. However, the dispersal limitation can also take place at fine scales [16,38]. Except for significant geographical barriers (such as river, mountain), the ability and mode of dispersal are also significant factors driving metacommunity composition, and those factors are more important when considering less active dispersers [11,45,46]. Soil mites are small and wingless animals that live in soil ecosystems. Active dispersal is the primary means for those soil-inhabiting animals to reach different localities with different qualities. The dispersal capacities of most soil mite species are low [47,48]. Moreover, the lack of continuous inter-connectance among soil pores might produce obstacles to the active movements of those soil mite species to some extent [49]. Actually, soil mite species were found to be severely dispersal limited even at an isolated distance as short as a few centimeters (5 cm) in a fragmented experiment [20], and the maximum active dispersal rate of those species was no more than 10 m per year [50,51]. Therefore, it might be difficult for soil mite species to track micro-environmental heterogeneity at the experimental scale.

In both years, soil mite metacommunities were significantly influenced by broad-scale and fine-scale MEM patterns. A broad-scale pattern indicates that large-scale spatial processes, such as dispersal limitation or spatially structured environmental gradients, play important roles in structuring the soil mite metacommunity [33,52]. Although the broad-scale MEMs were clearly related to environmental variables (mainly pH, moisture and SOM), the common fraction [g] that corresponded to the environmental and broad-scale variables was relatively low (0.54 and 0.58% in 2012 and 2013, respectively) (Fig 1). It was inferred that an important role was played by some unmeasured, underlying processes that are spatially structured and influence both the soil mite metacommunity and environmental factors (e.g., dispersal limitation at a large or regional scale) [32]. Otherwise, the fine-scale pattern implies that the soil mite metacommunity was most likely controlled by autocorrelated processes [53]. As an expression of the local spatial correlation generated by community dynamics [32], the fine-scale MEMs did not relate to any environmental variables, further emphasizing the importance of neutral biotic processes (such as random dispersal) [32].

According to the results of a variation partitioning analysis, purely environmental variables non-significantly explained little of the overall variation in metacommunity structure in both years. However, a positive contribution of environmental factors to metacommunity composition was detected in 2012 when controlling for spatial factors. Moreover, we found significant associations of the soil mite metacommunity with moisture (LWC and SWC) and soil organic matter (SOM), suggesting that both moisture condition and food availability have the potential to influence the soil mite metacommunity [22,37,38]. Meanwhile, to identify the roles of moisture and food availability on soil mite metacommunity constructuring, more manipulated experiments and precise technology (such as isotopic anlaysis) are suggested. However, these results can be interpreted as showing that without considering the combined contribution between spatial and environmental variables, it is difficult to neglect the environmental processes involved in the structure of the soil mite metacommunity at a small scale.

The contribution of spatial processes was more important than that of environmental processes for shaping the soil mite metacommunity on a small scale. The findings were consistent with the potential processes at a spatial extent of 5 × 5 m^2^ in the same temperate deciduous forest [38]. Although the underlying processes that drive community assembly are scale-dependent, the generality of possible processes shaping a soil mite metacommunity may be expressed when gradually upscaling from a fine scale (5 m) to a small scale (50 m) in the same habitat. Previously, much attention has been focused on the contribution of environmental gradients to soil mite metacommunity composition. However, there has not been a sufficient examination of the relative role of spatial variables in metacommunity structure. On a small scale in the study area, a spatial perspective should be incorporated to reveal the processes underlying soil mite community composition.

In this study, the soil mite metacommunity was primarily controlled by dispersal limitation on a broad scale and random dispersal on a fine scale (representing a verification of the neutral perspective) and was secondarily affected by environmental filtering (interpreted as species sorting) [46]. Therefore, the neutral and species sorting theories might simultaneously regulate the soil mite metacommunity at such a spatial scale, but the relative importance was asymmetric. However, it is difficult to clearly describe the influence of species sorting, mass effects and neutral perspectives in our experiment, as it is unclear whether the dispersal rate was large enough for successful colonization of other localities. In this study, the soil mite metacommunity may be regulated by a combination of several theory-relevant factors on a small scale [54]. One reason for the mixed results may be the use of a single diversity measure. We have suggested that functional and phylogenetic characteristics and differently weighted species similarities should be introduced to understand the drivers of a soil mite metacommunity [55].

## Conclusions

Our results suggested that the soil mite metacommunity was primarily constructed through dispersal limitation at a broad scale and by neutral biotic processes (such as random dispersal) at a small scale. Then, the contribution of environmental filtering was secondarily important, and soil moisture and soil organic matter were important. It was suggested that when evaluating the pattern and underlying processes of a soil mite metacommunity at small scale, the spatial processes should be considered carefully. These results emphasized a combination of neutral and species sorting perspectives in controlling the soil mite metacommunity at such a small scale.

## Supporting information

S1 Fig Sample-based rarefaction curves for the soil mite metacommunity in 2012 and 2013. The solid curves represent the means of the repeated re-sampling of all pooled species. The grey areas represent the 95% confidence limits of the curves
(TIF)

S2 Fig MEM eigenfunctions selected to model the soil mite metacommunity in 2012. The square bubble size is proportional to the value associated with it, whereas the color reflects the sign of the number (black = positive, white = negative).
(TIF)

S3 Fig MEM eigenfunctions selected to model the soil mite metacommunity in 2013. The square bubble size is proportional to the value associated with it, whereas the color reflects the sign of the number (black = positive, white = negative).
(TIF)

S1 Table Characteristics of environmental variables (n = 121 samples) in 2012 and 2013. (DOCX)

S2 Table Regression analysis correlation between the significantly canonical axes of broad-scale and fine-scale MEMs and environmental variables in 2012 and 2013. (DOCX)
