# Supplementary figures and images for: The underlying processes of a soil mite metacommunity on a small scale
Source: PLoS One. 2017 May 8;12(5):e0176828. doi: 10.1371/journal.pone.0176828 (PMC5421772; doi:10.1371/journal.pone.0176828)

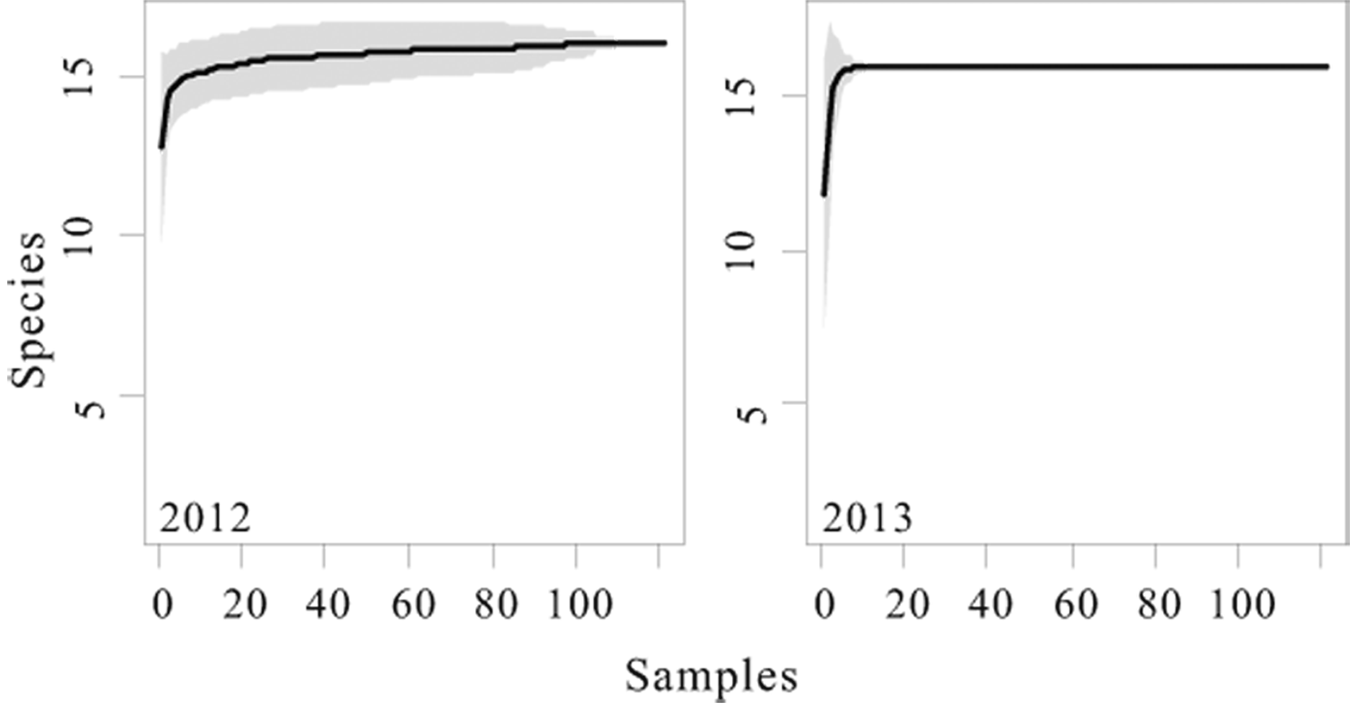

Supplement: S1 Fig — The solid curves represent the means of the repeated re-sampling of all pooled species. The grey areas represent the 95% confidence limits of the curves (TIF) [file pone.0176828.s001.tif]

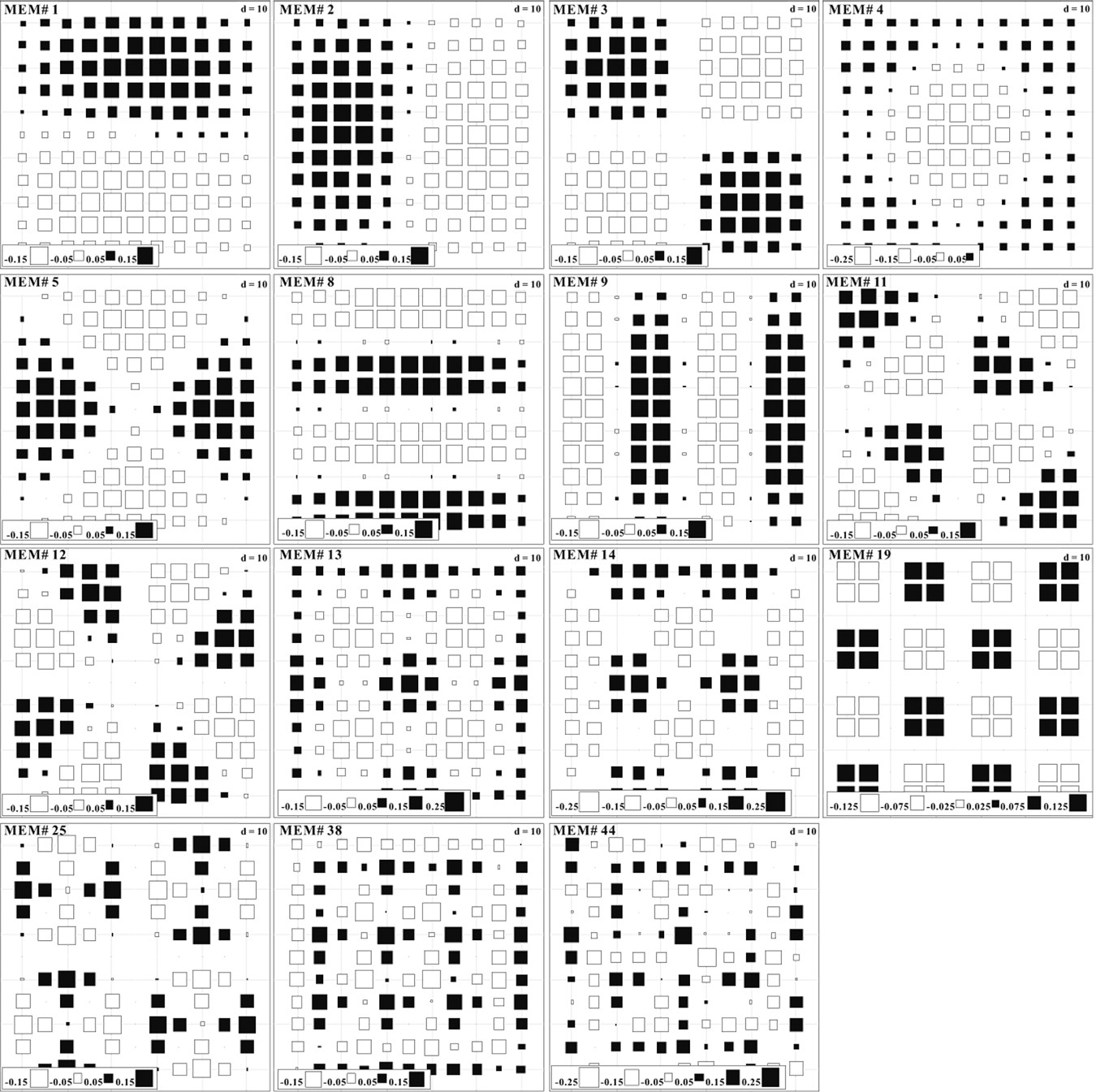

Supplement: S2 Fig — The square bubble size is proportional to the value associated with it, whereas the color reflects the sign of the number (black = positive, white = negative). (TIF) [file pone.0176828.s002.tif]

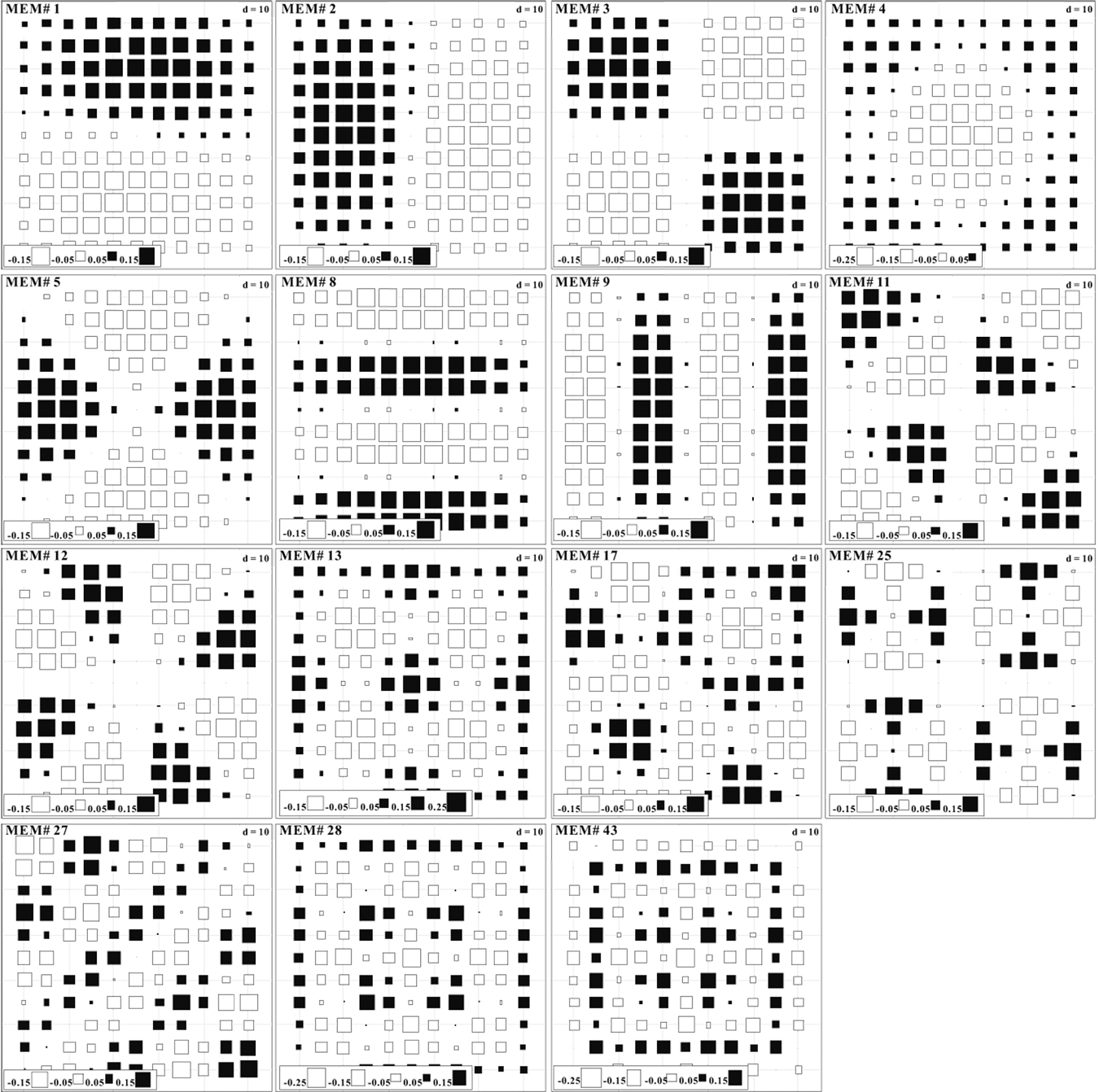

Supplement: S3 Fig — The square bubble size is proportional to the value associated with it, whereas the color reflects the sign of the number (black = positive, white = negative). (TIF) [file pone.0176828.s003.tif]
