# Supplementary material for: The underlying processes of a soil mite metacommunity on a small scale
Source: PLoS One. 2017 May 8;12(5):e0176828. doi: 10.1371/journal.pone.0176828 (PMC5421772; doi:10.1371/journal.pone.0176828)
Supplement: S1 Table — (DOCX) [file pone.0176828.s004.docx]

**S1 Table** Characteristics of environmental variables (n=121 samples) in 2012 and 2013.

|  | 2012 | | | | | 2013 | | | | |
| --- | --- | --- | --- | --- | --- | --- | --- | --- | --- | --- |
| Variable ^a^ | Mean | SD ^b^ | Kurtosis | Skewness | CV(%) ^c^ | Mean | SD | Kurtosis | Skewness | CV(%) |
| pH | 5.77 | 0.36 | -0.38 | 0.07 | 6.17 | 5.74 | 0.38 | -0.05 | 0.24 | 6.58 |
| SOM | 151.13 | 72.03 | 0.51 | 0.45 | 47.66 | 153.46 | 53.52 | -0.49 | -0.79 | 34.87 |
| SWC | 27.56 | 9.68 | 25.47 | 3.36 | 35.13 | 26.26 | 6.38 | -0.72 | 0.04 | 24.31 |
| LWC | 59.55 | 7.66 | 2.98 | 1.15 | 12.86 | 59.13 | 7.66 | 1.13 | 0.71 | 12.96 |
| LDW | 19.43 | 7.54 | 1.53 | 1.13 | 38.83 | 19.40 | 10.10 | 4.34 | 1.79 | 52.08 |

^a^ pH-soil pH, SOM-soil organic matter content (g kg^-1^), SWC-soil water content (%), LWC-litter water content (%) and LDW-litter dry weight (g).

^b^ SD-standard deviation.

^c^ CV- coefficient of variation.
