# Supplementary material for: The underlying processes of a soil mite metacommunity on a small scale
Source: PLoS One. 2017 May 8;12(5):e0176828. doi: 10.1371/journal.pone.0176828 (PMC5421772; doi:10.1371/journal.pone.0176828)
Supplement: S2 Table — (DOCX) [file pone.0176828.s005.docx]

**S2 Table** Regression analysis correlation between the significantly canonical axes of broad-scale and fine-scale MEMs and environmental variables in 2012 and 2013.

|  | 2012 | | | | | | 2013 | | | | | | | |
| --- | --- | --- | --- | --- | --- | --- | --- | --- | --- | --- | --- | --- | --- | --- |
| Environmental variables ^b^ | Broad-scale | | | | | Fine-scale | Broad-scale | | | | | | | Fine-scale |
|  | RDA1^a^ | RDA2 | RDA3 | RDA4 | RDA5 | RDA1 | RDA1 | RDA2 | RDA3 | RDA4 | RDA5 | RDA6 | RDA7 | RDA1 |
| PC1 | -0.96 | -2.82^**^ | 2.49^**^ | 1.33 | 1.89 | -0.59 | 4.31^***^ | -2.57^*^ | -0.82 | 1.55 | -1.04 | -0.41 | -1.11 | 0.19 |
| PC2 | -0.42 | 0.93 | -2.20^*^ | -2.01^*^ | -1.80 | 1.47 | 0.66 | 1.64 | 0.44 | 0.79 | -0.51 | 0.83 | 2.93^**^ | 0.46 |
| PC3 | 0.68 | -0.74 | -0.67 | 0.77 | -2.36^*^ | 0.36 | -3.11^**^ | -1.07 | 0.66 | -1.97 | 0.45 | -0.59 | -1.21 | -0.34 |
| PC4 | -2.40^*^ | -1.29 | 1.26 | -1.66 | -1.13 | 0.49 | 1.96 | 2.64^*^ | -1.78 | 0.43 | -0.47 | 1.03 | 0.71 | 0.44 |

^a^ The significantly canonical axes of broad-scale and fine-scale MEMs based on RDA.

^b^ PC indicates each of the environmental factors that was obtained from the PCA (principal component analysis).

^*^ *P*<0.05. ^**^ *P*<0.01. ^***^ *P*<0.001.
